# Integrated Metabolomics and Transcriptome Analysis of Anthocyanin Biosynthetic Pathway in *Prunus serrulata*

**DOI:** 10.3390/plants14010114

**Published:** 2025-01-03

**Authors:** Qi Ye, Feng Liu, Kai Feng, Tao Fu, Wen Li, Cheng Zhang, Meng Li, Zhilong Wang

**Affiliations:** 1 Department of Horticultural Technology, Ningbo City College of Vocational Technology, Ningbo 315000, China; yeqi0206@outlook.com (Q.Y.); coy24@163.com (F.L.); fengkai@njfu.edu.cn (K.F.); futao@nbcc.cn (T.F.); liwen547249317@126.com (W.L.); 2 College of Biology and the Environment, Nanjing Forestry University, Nanjing 210037, China; chengz@njfu.edu.cn

**Keywords:** *Prunus*, cherry, transcriptome, anthocyanin biosynthetic pathway

## Abstract

*Prunus serrulata* is an important landscape tree species whose flower color has high ornamental value. However, the molecular mechanisms regulating flower color in *P. serrulata* remain unclear. By studying the metabolomics and transcriptomics of three different color varieties under the species lineage of *P. serrulata*, ‘Eigeng’ (EG, white), ‘Albo-rosea’ (AR, pink), and ‘Grandiflora’ (GF, green), the biosynthetic mechanisms of different flower colors in *P. serrulata* were revealed. The results showed that the different colors of the petals were related to the content of chlorophyll and anthocyanins. Among these, cyanidin-3-O-glucoside and cyanidin-3-O-(6-O-malonyl-β-D-glucoside) were highly expressed in AR. A combined transcriptomic analysis revealed that five flavonoid structural genes, including two *DFR* genes and three *UFGT* genes, were specifically expressed. In addition, three key transcription factors, *PsMYB77*, *PsMYB17*, and *PsMYB105*, were identified as regulators of the structural genes *DFR* and *UFGT* and participants in the forward synthesis of anthocyanin. This study provides convincing evidence elucidating the regulatory mechanisms of anthocyanin synthesis of *P. serrulata* and provides a theoretical basis for the breeding and development of new varieties and germplasm resource innovation for cherry blossom.

## 1. Introduction

Flowers’ color has significant biological importance for plants and serves as a crucial ornamental trait in evaluating the value of tree species in landscaping [1]. Color differences are related to the type and content of pigments, which can be divided into three categories: flavonoids, carotenoids, and alkaloids. Among these, anthocyanins are the most important flavonoid pigments and are widely found in angiosperms such as *Prunus pseudocerasus* [2], *Cymbidium* [3], *Rosa rugosa* [4], and *Rhododendron sanguineum* [5]. At present, the known anthocyanins are mainly divided into delphinidin, peonidin, pelargonidin, cyanidin, petunidin, and malvidin. These pigments aid in seed spread and reduce the negative effects of environmental factors such as ultraviolet light, low temperature, and drought on plants [6]. Additionally, anthocyanins exhibit strong antioxidant properties and other health-promoting functions [7].

The structural genes encoding multiple enzymes in the anthocyanin biosynthesis pathway are divided into two categories: upstream genes, also known as early synthetic structural genes (EBGs), including *PAL*, *CHS*, *CHI*, *F3H*, *F3’H,* and *F3’5’H*, and downstream genes, referred to as late synthetic structural genes (LBGs), such as *DFR*, *ANS*, *UFGT,* and *GT*. Studies have shown that EBGs exhibit high activity during the early stage of plant growth and development, while the accumulation of LBGs significantly increases during the later stage of maturation [8]. Anthocyanin biosynthesis is regulated by the interaction of specific transcription factors from the MYB and bHLH families and the WD40 protein. Among these, MYB is the largest transcription factor family in plants, controlling multiple metabolic pathways, such as secondary metabolism, growth and development, and signal transduction [9]. MYB transcription factors positively regulate the synthesis of flavonoid pigments in cash crops such as grapes, apples, and strawberries [10,11,12]. However, the specific regulatory mechanism for anthocyanin remains unclear, and whether there are novel regulatory factors and mechanisms involved in flower color variation still needs to be further studied.

*Prunus serrulata* is widely distributed in the western and eastern regions of China and has also been recorded on the Korean Peninsula and in Japan [13,14,15,16]. The species is well adapted to diverse geothermal conditions [17]. With a wide variation in its traits and flower color variation, *P. serrulata* serves as the parent of many ornamental cherry species and is a very important germplasm resource for flowering species [18]. Utilizing *P. serrulata* as a parent, a variety of natural and artificial hybrids have been developed and selected [19]. The current research on *P. serrulata* has focused on systematic classification [20,21,22], phylogeography [23], and in vivo tissue culture [24], and genome sequencing of *P. serrulata* has been completed [16]. Although flower color is an important ornamental trait of cherry blossoms, the metabolic pathways and regulatory mechanisms associated with flower color variation in *P. serrulata* varieties remain unexplored.

We aimed to explore the differential coloring substances in various flowers and identify the key transcription factors and differential genes in the anthocyanin synthesis pathway, discover new mechanisms of flower color regulation, and enhance our theoretical understanding of flower color regulation. Furthermore, our study provides a theoretical basis for subsequent flower color improvements and germplasm resource innovation.

To study the regulatory mechanisms of the flower color of different varieties of *P. serrulata*, we selected AR (*P. serrulata* ‘Albo-rosea’, pink) and GF (*P. serrulata* ‘Grandiflora’, green) as the research materials, with EG (*P. serrulata* ‘Eigeng’, white) as the control. By integrating a metabolome and transcriptome analysis, we aimed to explore the differentially colored substances in various flowers and identify key transcription factors and differential genes in the anthocyanin synthesis pathway. This study sought to elucidate the molecular mechanisms underlying flower color formation in *P. serrulata*, discover new mechanisms of flower color regulation, and enrich our theoretical understanding of flower color regulation. At the same time, it provides a theoretical reference for the subsequent improvement of flower color and germplasm resource innovation.

## 2. Results

### 2.1. Pigment Content Analysis

**Figure 1:**
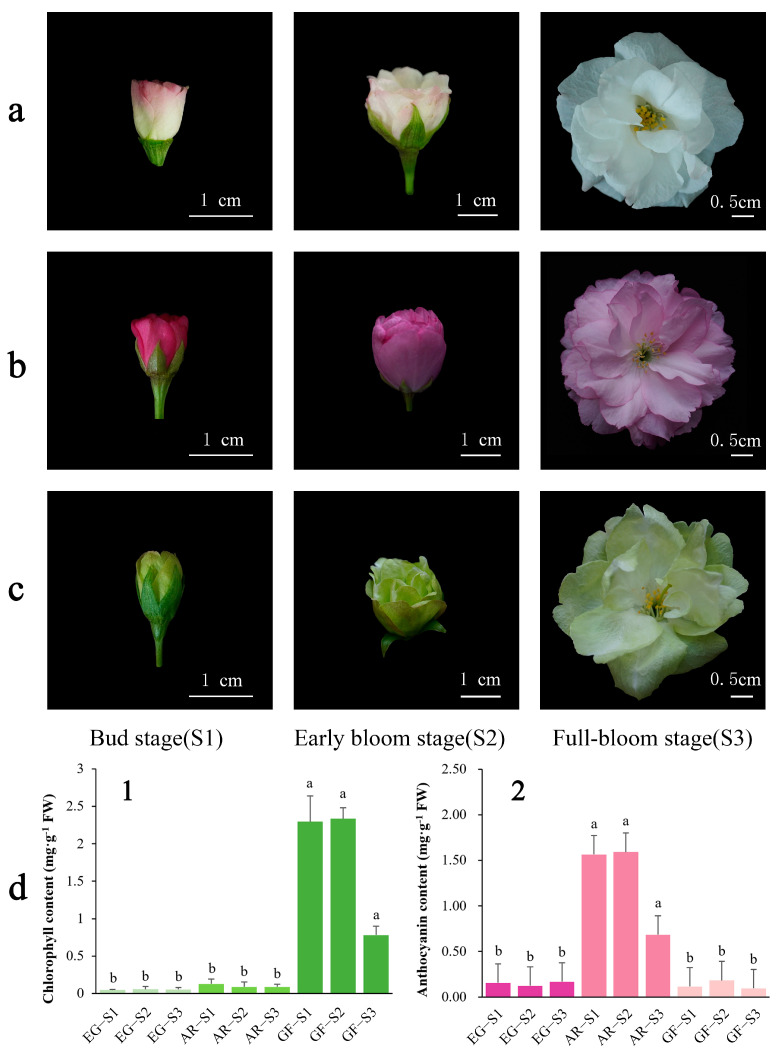
Phenotypes and pigment content of three flowering stages of different cultivars of *P. serrulata*: (**a**) ‘Eigeng’ (EG); (**b**) ‘Albo-rosea’ (AR); and (**c**) ‘Grandiflora’ (GF). (**d1**): Contents of total chlorophyll; (**d2**): contents of total anthocyanin. Lowercase letters indicate significant differences in the anthocyanin and chlorophyll content between cultivars (*p* < 0.05).

The color difference between different cultivars is large, and there are obvious differences by period (Figure 1a–c). The pigment content of the petals was determined in the full flowering period. The chlorophyll content of GF is much higher than that of the other two varieties, and with a decrease in the chlorophyll content, the color of the petals gradually becomes lighter in the full flowering period. The anthocyanin content of AR was higher than that of the other varieties (Figure 1d), so it was inferred that the main reason for the green color of GF was chlorophyll deposition. Previous studies have shown that anthocyanins play a key role in plant color. Combined with existing phenotypes and physiological indicators, anthocyanins were selected for further targeted metabolomics detection.

### 2.2. Metabolome Profiling of Petal Samples in Three Cultivars

The anthocyanin content in the different cultivars was determined using an UPLC-MS platform. Three biological replicates were carried out, and qualitative and quantitative analyses were carried out. A total of 42 anthocyanin-related metabolites were identified, including 7 petunidins, 6 delphinidins, 6 flavonoids, 1 malvidin, 4 peonidins, 8 cyanidins, 7 pelargonidins, and 3 procyanidins (Table S1). Clustering of the metabolites (Figure S1) showed good biological repeatability and consistent trends in the expression within the sample group. There were significant differences between the cultivars, and AR had obvious separation from the other two groups.

**Figure 2:**
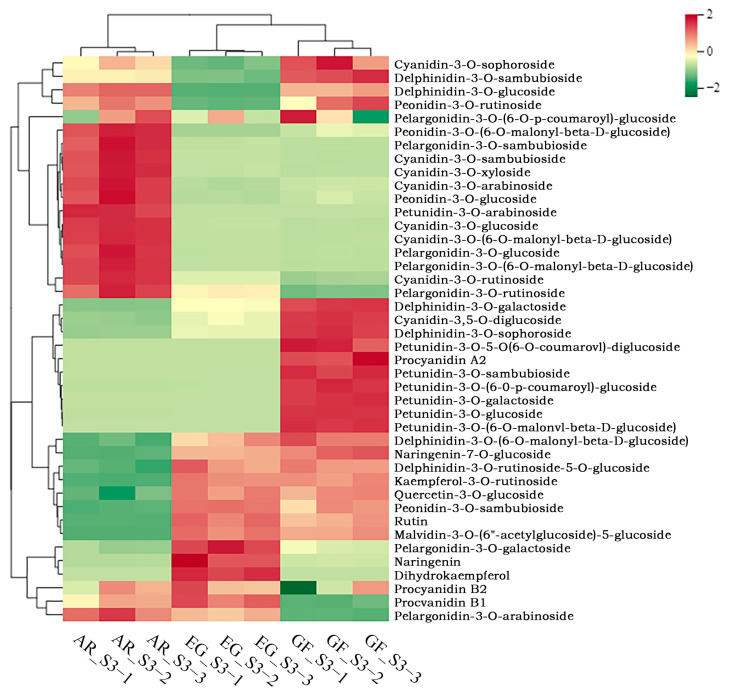
Heat map clustering showing the correlation between the pink, white, and green flower samples based on 42 metabolite profiles. AR: *P. serrulata* ‘Albo-rosea’, pink flower; GF: *P. serrulata* ‘Grandiflora’, green flower; EG: *P. serrulata* ‘Eigeng’, white flower. The color scale from red to green in the heat map represents the normalized metabolite contents.

**Figure 3:**
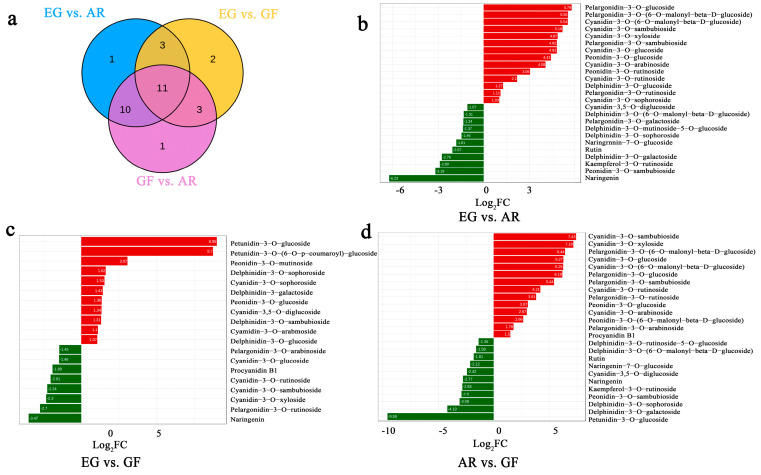
Analysis of different metabolites of three cultivars of *P. serrulata*. (**a**) Venn diagram of differential metabolites; (**b**–**d**) bar chart of differential metabolites, with red indicating increased metabolites and green indicating reduced metabolites.

To explore the differences in the anthocyanins in the blooming stage of the three different varieties, 32 anthocyanins and flavonoids were standardized for their metabolite content, and then a cluster heat map was drawn (Figure 2). There were significant differences between the samples, and the production of cyanidin in AR was higher than that in the other two varieties. A total of 10 common differentially accumulated metabolites (DAMs) were screened for differential anthocyanin metabolites based on them having a *p*-value ≤ 0.5 (Figure 3, Table S2), namely cyanidin-3,5-O-diglucoside, cyanidin-3-O-arabinoside, cyanidin-3-O-glucoside, cyanidin-3-O-rutinoside, cyanidin-3-O-sambubioside, cyanidin-3-O-xyloside, pelargonidin-3-O-rutinoside, peonidin-3-O-glucoside, cyanidin-3-O-sambubioside, cyanidin-3-O-xyloside, delphinidin-3-O-galactoside, and delphinidin-3-O-sophoroside. The differences in the quantitative information on the metabolites in each group were compared, and the top 20 metabolites were selected to draw a bar chart of their differential metabolisms. Cyanidin-3-O-glucoside and cyanidin-3-O-(6-O-malonyl-beta-D-glucoside) were highly expressed in AR, while Petunidin-3-O-glucoside was significantly up-regulated in EG and GF. This shows that cyanidin plays an important role in the process of pink coloration.

**Figure 4:**
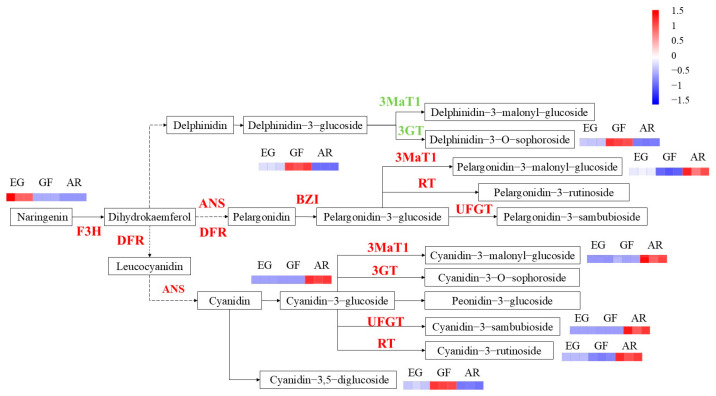
Anthocyanin biosynthesis pathway of *P. serrulata* cultivars. Note: The red and green letters indicate higher and lower expression levels of genes in *P. serrulata*.

The differential metabolites were further mapped as differential pathways (Figure 4, Table S3). Naringin forms dihydrokaempferol under the action of F3H, which is the substrate of the key downstream enzyme DFR. The high expression of the DFR enzyme generates leucocyanidin, which further forms cyanidin and pelargonidin, two pigments up-regulated in expression in pink petals. Through key enzymes such as RT, UFGT, BZI, and GT, key downstream anthocyanidins such as cyanidin-3-glucoside and pelargonidin-3-glucoside are formed, which ultimately affect the coloration of AR’s petals.

### 2.3. Transcriptome Sequencing and Analysis

A total of 183.91 Gb of clean reads was obtained from 27 cDNA libraries (with three biological replicates per sample) after quality control and filtration. The percentage of Q30 bases in all of the samples was above 93.34%, the GC content was between 43.81% and 45.49%, and 4385 new genes were detected and annotated, indicating a low overall data error rate and a high sequencing quality (Table S4). The comparison efficiency of the total reads was higher than 70%, and 71.27% of the data was compared to the exon region, indicating a high utilization rate of the transcriptome data and a good match with the reference genome for *P. serrulata* (Table S5). A total of 29,839 gene sequences were obtained after assembly.

### 2.4. Differentially Expressed Genes in Three Cultivars

**Figure 5:**
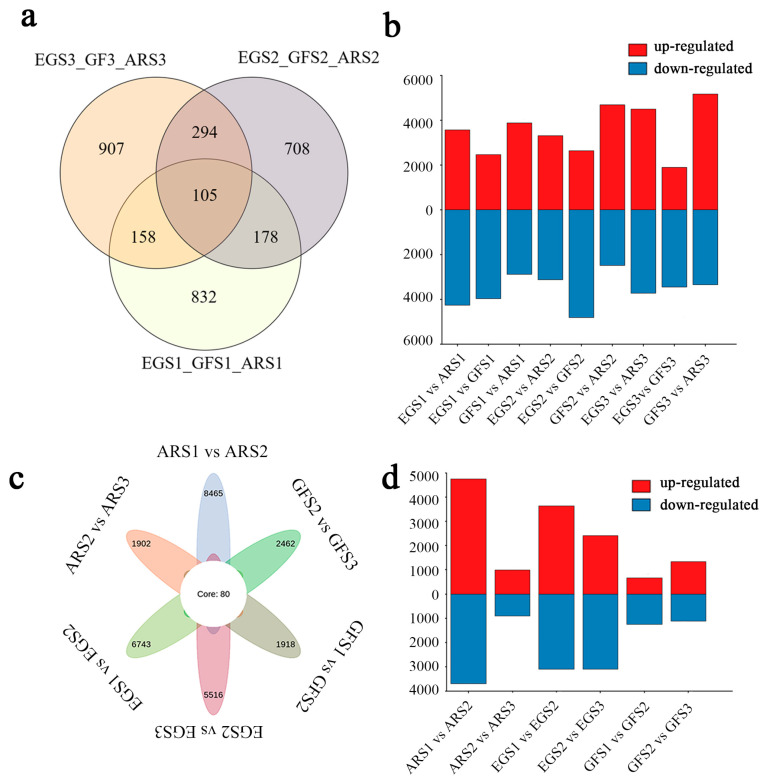
Differential genes of *P. serrulata* cultivars. (**a**) Different genes between cultivars; (**b**) up- and down-regulated genes at different developmental stages; (**c**) differential genes at different developmental stages; (**d**) up- and down-regulated genes at different developmental stages.

To analyze the specific molecular mechanisms of floral color differences, genes with a |log2Fold Change| ≥ 1 and an FDR < 0.05 were selected as differentially expressed genes, and a differential gene expression analysis was performed using DESeq2. A total of 16,019 DEGs were detected between groups (Figure S2, Table S6). The color of the petals of different varieties at the same growth and development stage is obviously different. DEGs from the same period were screened, including 1273 DEGs from the S1 period, 1285 DEGs from the S2 period, and 1464 DEGs from the S3 period. Among them, GFS3 and ARS3 had the most differential genes, with 8528 differential genes and 105 common DEGs after screening (Figure 5a,b, Table S7). Secondly, the petal color in the same variety gradually faded, and the differential genes among neighboring samples were screened, and 80 common DEGs were finally obtained (Figure 5c,d, Table S7). The common conserved genes may be related to flower color variation. After removing duplicate genes from the different varieties of DEGs and DEGs at different developmental stages, 13,572 genes were used for the follow-up analysis.

**Figure 6:**
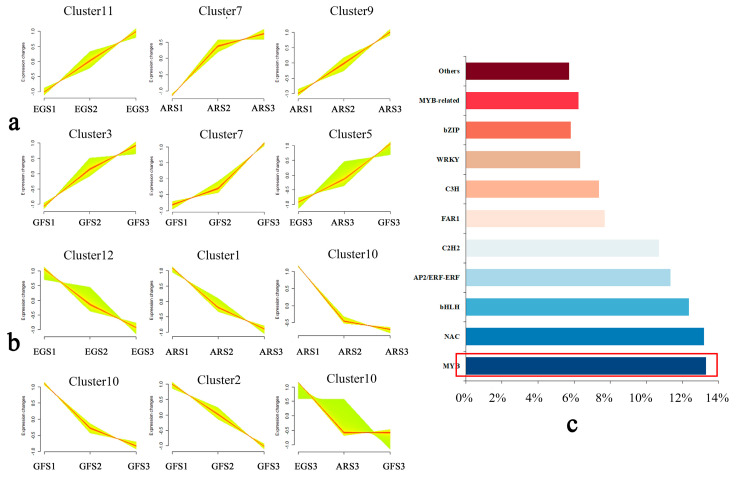
Differential gene K-means cluster and transcription factor statistics. (**a**) Up-regulated genes; (**b**) down-regulated genes; (**c**) transcription factor number statistics.

A K-means cluster analysis of the differential genes using the FPKM values showed that 12 gene clusters were consistent with the phenotypic trends (Figure 6, Table S8). The up-regulated expression of the differential gene clusters Cluster7 and Cluster9 in the same variety may interact with transcription factors to make pink petals pale. The expression of 712 genes from Cluster5 increased significantly in AR during the S3 period. These genes encode a variety of transcription factors, including MYB306 and WD40. Structural genes related to anthocyanins, such as *CHI* and *UFGT*, are also included. In the transcription factor statistics on the gene expression clusters, MYB and MYB-related differential genes accounted for the highest proportion (19.6%). In Cluster10, which was down-regulated among the varieties, the expression trend for most of the MYB transcription factors was down-regulated in AR, such as MYB113 and MYB114. Subsequent studies focused on an analysis of the relevant transcription factors in this cluster, combined with previous studies, concluded that MYB may be involved in the regulation of flower color in *P. serrulata*.

### 2.5. The Chlorophyll Biosynthesis Pathway During Flower Color Changes

**Figure 7:**
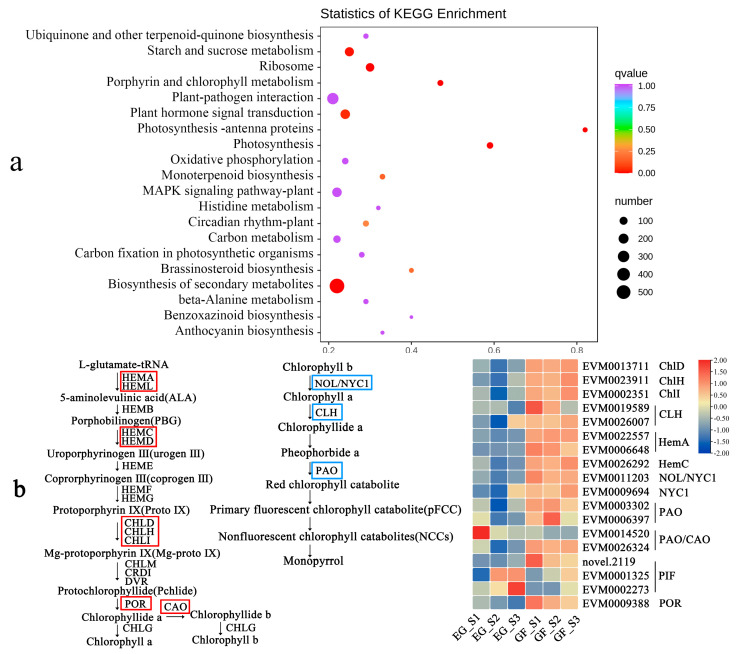
KEGG enrichment analysis of EG and GF differential genes. (**a**) Pathway enrichment; (**b**) chlorophyll metabolism pathway and heat map.

In the contrast group of EG and AR, a total of 2136 differential genes were annotated and 187 metabolic pathways were enriched, including porphyrin and chlorophyll metabolism, the biosynthesis of secondary metabolites, and other pathways (Figure 7a). The content of chlorophyll in the GF petals was very high in the previous determination (Figure 1d), focused on genes related to chlorophyll synthesis and metabolism. A total of 15 DEGs participating in the up-regulated expression of chlorophyll synthesis were screened. The expression of two *NYC1*, two *CLH*, and two *PAO* DEGs related to chlorophyll degradation was up-regulated in GF (Figure 7b, Table S9).

### 2.6. The Candidate Genes Involved in the Anthocyanin Biosynthesis Pathway

**Figure 8:**
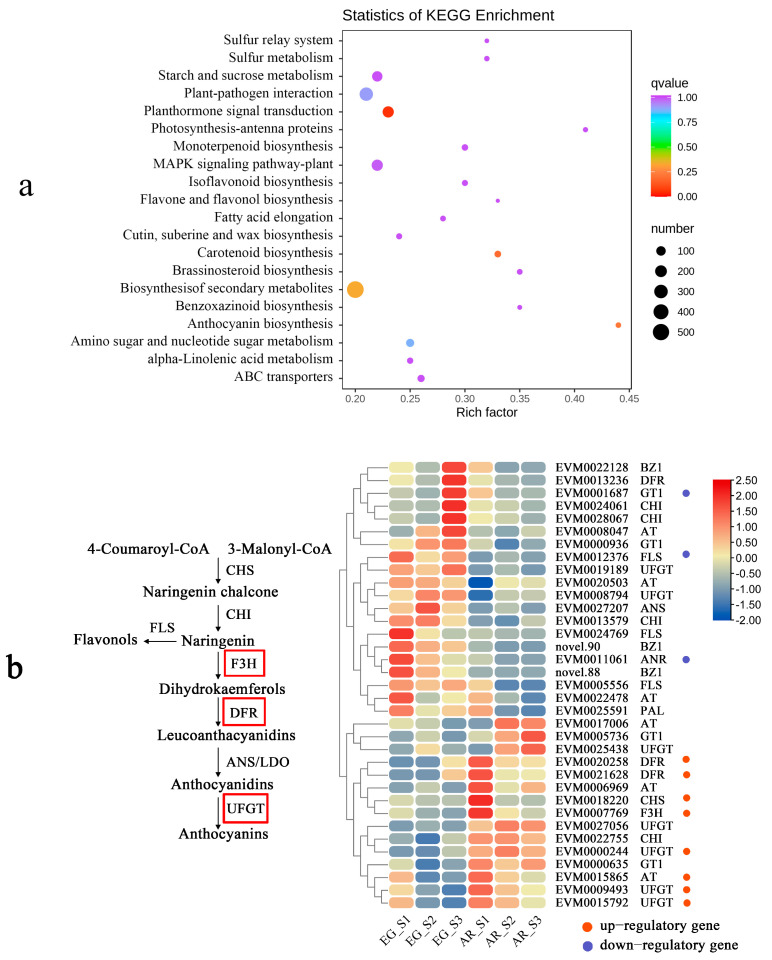
KEGG enrichment analysis of EG and AR differential genes. (**a**) Pathway enrichment; (**b**) anthocyanin metabolism pathway and heat map.

In the contrast group of EG and AR, a total of 2101 differential genes were annotated and 196 metabolic pathways were enriched, and 4 pathways were significantly enriched (*p* < 0.05). These were plant hormone signal transduction, carotenoid biosynthesis, anthocyanin biosynthesis, and the biosynthesis of secondary metabolites (Figure 8a). The key enzyme genes related to anthocyanin biosynthesis were screened. There were 11 DEGs encoding enzymes related to anthocyanin metabolism; 3 genes were down-regulated, and 8 genes were up-regulated (Figure 8b, Table S10). After an analysis of the expression patterns, it was found that the expression patterns of eight genes were consistent with the expression patterns of AR. These included *DFR* genes (EVM0020258 and EVM0021628), *UFGT* genes (EVM0000244, EVM0009493, and EVM0015792), a *CHS* gene (EVM0018220), and an *F3H* gene (EVM0007796). The expression levels of these eight genes were low in EG but high in AR, and the expression levels gradually decreased from S1 to S3. The high expression of these eight genes may be the reason for the high content of total anthocyanins in AR.

### 2.7. MYB Transcription Factors Related to Anthocyanin Synthesis in P. serrulata

**Figure 9:**
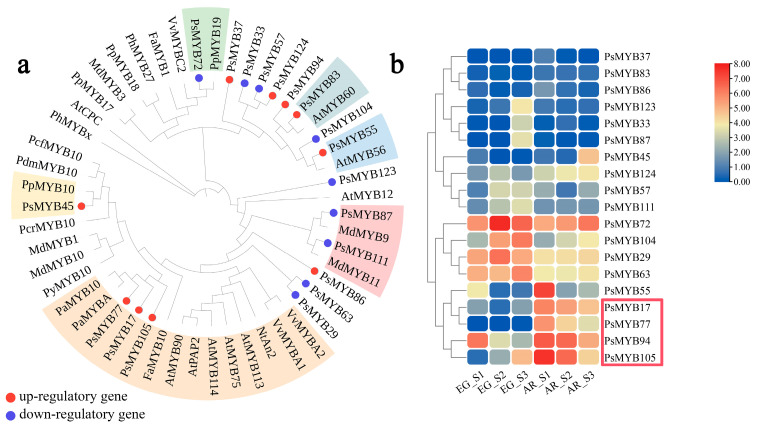
Analysis of anthocyanin regulation DEGs among cultivars of *P. serrulata*. (**a**): Phylogenetic tree; (**b**): differential expression heat map.

Multiple sequences were compared with EG, and 19 R2R3-MYB transcription factors were obtained. A phylogenetic analysis of the identified MYB transcription factors with Arabidopsis and other proximal species was performed. Among the differential genes, 9 genes were down-regulated and 10 genes were up-regulated (Figure 9, Table S11). Among these, *PsMYB72* was clustered in a branch with *PpMYB19*, an anthocyanin gene known to be down-regulated in the same genus, in *P. persica.* This gene is highly expressed in EG, and its expression increases as AR’s flower color becomes lighter. It may down-regulate the synthesis of flower color in *P. serrulata*. Three genes, *PsMYB77*, *PsMYB17,* and *PsMYB105,* were up-regulated; likewise, *AtMYB113* and *AtMYB114* in the S6 subfamily, which are related to the positive regulation of anthocyanins in *Arabidopsis thaliana*, were clustered into one branch. The expression trends of 19 MYB transcription factors were analyzed further. The expression trend for *PsMYB77*, *PsMYB17,* and *PsMYB105* was consistent with the content of anthocyanins and the expression trend for *DFR* (EVM0020258 and EVM0021628) and *UFGT* (EVM0000244, EVM0009493, and EVM0015792) in the structural genes. Combined with the results of the phylogenetic tree and trend analyses, which suggested that *PsMYB77*, *PsMYB17,* and *PsMYB105* may be involved in the positive regulation of anthocyanin biosynthesis. *PsMYB72* may negatively regulate flower color synthesis in *P. serrulata*. The qRT-PCR results of gene quantitative verification were similar to those of RNA-seq, indicating the accuracy and reliability of the results of the analysis.

### 2.8. qRT-PCR Validation of the Expression Patterns in Anthocyanin-Related Genes

**Figure 10:**
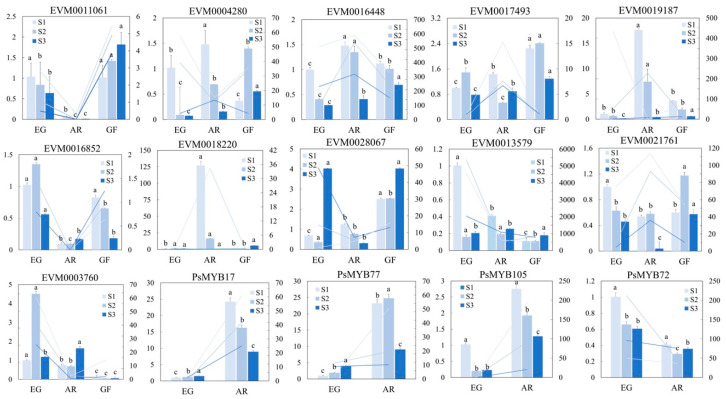
qRT-PCR verification of partial structural and MYB genes. Note: Bar chart: q-PCR content of different cultivars; line chart: RNA content of different cultivars. Lowercase letters indicate significant differences in the qPCR results between cultivars in the same time period (*p* < 0.05).

In order to verify the reliability of the results of RNA-seq, 11 structural genes and 4 transcription factors related to anthocyanin synthesis were selected in this study. qRT-PCR was used to verify the expression patterns of these genes, and the quantity of the gene expression in EG was used as a reference. The results showed that the overall qRT-PCR expression trends in these genes were basically consistent with the transcriptome results. The transcriptome results were relatively reliable (Figure 10).

## 3. Discussion

### 3.1. Effects of Pigment Content and Types on Flower Color in P. serrulata

Flower color is an important ornamental trait to measure the value of horticultural tree species, and it is an adaptive phenotype in the process of natural evolution. In recent years, the mechanism of flower color has been the focus of biological research, and species and plant pigment content are important factors that affect color diversity [25,26,27]. The flower color differences among *Rosa rugosa* varieties in full flowering were caused by different cyanidin contents [26]. Flower appearance in *Rhododendron simsii*, *Paeonia lactiflora*, and *Trifolium repens* was also related to anthocyanins [28,29,30]. In this study, the total anthocyanin content of the three varieties was determined, and it was found that the anthocyanin content of AR was much higher than that of other varieties, and further qualitative and quantitative analyses were needed. Chlorophyll is widely studied in relation to plant leaf color, and the expression of the gene for chlorophyll decomposition is down-regulated in *Pennisetum setaceum*, resulting in leaf chlorosis [31]. The chlorophyll content of GF was higher than that of the other two varieties in all periods, and the content decreased with the flowering process, which proved that chlorophyll was an important pigment for its green appearance, and further research on its synthetic pathway was needed.

Cyanidin and its derivatives are widely active in red flower petals in plants [32]. Du and Liu studied the metabolites of 30 species of *Rhododendron* with different colors and found that the main compound in the red variety was cyanidin [33,34]. A total of 42 anthocyanins in seven categories were detected in this study. Cyanidin and pelargonidin were significantly up-regulated in AR, which was consistent with the results of previous studies, indicating that cyanidin played an important role in the color of pink petals. Further screening of 11 key DAMs was undertaken, and the content of cyanidin-3-O-rutinoside was the highest in AR, which may be the key metabolite for color. Wang and Li both found that cyanidin was significantly up-regulated in different color varieties of safflower and orchid in their studies on color differences [35,36], which proved that the metabolomics results of this study were reasonable to a certain extent.

### 3.2. The Key Genes Involved in Anthocyanin Biosynthesis in P. serrulata

Anthocyanin content and species differences have direct effects on flower color. In recent years, anthocyanin synthesis genes in flowering plants have been extensively studied. Studies on the structural genes in *Yulania denudata*, *Camellia japonica*, rose, and other ornamental plants have found that the expression levels of the *F3H*, *CHI*, *DFR,* and *UFGT* genes are strongly correlated with anthocyanin content [4,37,38,39]. In this study, 35 key structural genes related to anthocyanin biosynthesis were activated in AR, including *CHS*, *FLS*, and *F3H* upstream and *ANR*, *ANS*, *UFGT*, and *DFR* downstream. Studies have shown that decreased activity of the *ANS* and *DFR* genes can lead to anthocyanin degradation [40]. *DFR* is the first key enzyme downstream of anthocyanins. In studies on the flower color of *Cymbidium sinense* and *Paeonia ostii* petals, it was found that the *DFR* and *UFGT* genes played a decisive role in anthocyanin synthesis [41,42]. As the final step in the flavonoid pathway, the *UFGT* gene converts anthocyanins into a more stable water-soluble state [43]. In this study, genes encoding the *DFR* and *UFGT* enzymes were up-expressed in AR. EVM0000244, EVM0009493, and EVM0015792 were related to UDP-glucose-fructose-phosphate glucosyltransferase, and EVM0020258 and EVM0021628 were related to cinnamoyl-CoA enzymes, and the expression levels of five genes decreased with the increase in flowering time during the S1–S3 period. This is consistent with the metabolomic inference referred to in the previous section. It is concluded that the expression of the *DFR* and *UFGT* genes plays a key role in the formation of pink petals.

### 3.3. The Key Genes Involved in Chlorophyll Biosynthesis in P. serrulata

Sakuraba found that *POR* is an important gene for chlorophyll synthesis in *Oryza sativa*. In yellow leaf varieties of *Lagerstroemia indica*, the *HemA* and *HemD* genes are down-regulated, resulting in leaf cyanosis [44]. After the analysis of the differential genes in this study, the *HemA*, *HemC*, *ChlD*, *ChlH*, *ChlI*, *POR*, and *CAO* genes were found to be up-regulated in GF, and the differential expression of these genes was related to chlorophyll synthesis. Sato studied the aging process of rice and found that the mutation of the *NOL* and *NYC1* genes inhibited the degradation of chlorophyll b [45]. *AtCLH1* and *AtCLH2* in the leaves of *Arabidopsis thaliana* were related to chlorophyll degradation [46]. In this study, two *NYC1*, one *PAO*, and two *CLH* enzyme genes related to chlorophyll degradation were up-regulated in GF, and these genes were related to the green color of the petals, suggesting that the interaction of multiple genes may form a complex regulatory mechanism that ultimately affects the green color in petals.

### 3.4. Transcription Factors Related to Anthocyanin Biosynthesis

MYB is the largest transcription factor family in plants, and the anthocyanin biosynthesis pathway is regulated by highly conserved MYB, which is widely involved in the growth, development, and secondary metabolism of plants [47]. In this study, 124 MYB genes related to anthocyanin regulation were identified in cherry blossom, and the number of gene families was similar to that in plum blossom, peach blossom, and crabapple, indicating that closely related species have similar gene family structures [48]. Previous studies have shown that transcription factors from the S4, S5, S6, and S7 subfamilies in Arabidopsis thaliana are involved in plant flavonoid biosynthesis [49,50,51,52]. The S4 family is involved in the regulation of structural genes that inhibit anthocyanin expression, and the S5, S6, and S7 subfamilies are involved in the biosynthesis of proanthocyanins, anthocyanins, and flavonoids, respectively. In this study, six differential genes were clustered into the subfamilies S4 (*PsMYB72*), S6 (*PsMYB17*, *PsMYB45*, *PsMYB77*, and *PsMYB105*), and S7 (*PsMYB86*). In the key S6 subfamily, three R2R3-MYB genes, *PsMYB77*, *PsMYB17,* and *PsMYB105*, were clustered together into one branch, and their expression trend was consistent with that of the metabolome and structural genes, suggesting that they play a key role in the positive regulation of anthocyanin synthesis.

## 4. Materials and Methods

### 4.1. Plant Materials and Sampling

The white flower *P. serrulata* ‘Eigeng’ (EG), pink flower *P. serrulata* ‘Albo-rosea’ (AR), and green flower *P. serrulata* ‘Grandiflora’ (GF) were cultivated at the Longshan Cherry Garden (118°01′46″ E, 32°02′46″ N), located in Chuzhou, Anhui province, China. Samples of petals at different stages of development were collected from different directions of the tree and mixed on 25 March 2021. Three biological replicates were gathered per sample. The petal samples were immediately frozen in liquid nitrogen and stored at −80 °C for subsequent transcriptomic and metabolomic analyses.

### 4.2. Measurement of Chlorophyll and Total Anthocyanin Contents

Chlorophyll was extracted as described by Li. et al. with some modifications [53]. We added 15 mL of 95% ethanol to a 0.1 g petal sample and extracted it for 24 h in a dark environment. The absorbance of the solution was measured at 665 nm and 649 nm. The chlorophyll a (Ca) and chlorophyll b (Cb) contents were calculated using the formulae Ca = (13.95 × A665 − 6.88 × A649) and Cb = (24.96 × A649 − 7.32 × A665). A 0.1 g sample was added to 600 μL of anthocyanin extract (CH_4_O:H_2_O:HCl = 80:20:1) and extracted for 2 h at 4 °C. Then, we added 400 μL of distilled water and CHCl_3_, centrifuged it at 12,000× *g* for 10 min, and took the supernatant. The absorbance of the solution was measured at 530 nm and 657 nm. The total anthocyanin content was calculated using the following formula: Q (mg·g^−1^FW) = (A_530_ − 0.25 × A_657_)·g^−1^. Three biological replicates were performed for each sample.

### 4.3. Sample Preparation and Extraction

Anthocyanin metabolites were extracted following the previous protocol by Boulton with some modifications [26]. The samples were freeze-dried and ground into powder and extracted with methanol/water/hydrochloric acid. Then, the extract was vortexed in an ultrasound for 5 min and centrifuged. The residue was re-extracted by repeating the above steps again under the same conditions. The supernatants were collected and filtrated through a membrane filter before the LC-MS/MS analysis. The anthocyanin extraction was based on Ferrars et al. [27]. The data acquisition instruments included the use of UPLC and MS/MS. The liquid-phase conditions included the following: (1) An ACQUITY BEH C18 1.7 µm, 2.1 mm, × 100 mm column. (2) Mobile phase A: Ultrapure water (0.1% formic acid). Mobile B phase: Methanol (0.1% formic acid). (3) The elution gradient was set to 6 min at 5% B phase; 6 min at 50% B phase; and 2 min at 95% B phase, kept for 2 min, decreased to 5% at 14 min, and equilibrated for 2 min. (4) Flow rate: 0.35 mL per minute. Column temperature: 40 °C. The injection volume was 2 μL. The Ms conditions included the following: Electrospray ion source (ESI) temperature: 550 °C. MS voltage: 5500 V in positive ion mode. Curtain gas (CUR): 35 psi. In the Q-Trap 6500+, each ion pair was scanned according to the optimized declustering potential (DP) and collision energy (CE).

### 4.4. Anthocyanin Identification, Quantification, and Data Analysis

Anthocyanin identification and quantification were carried out using MetWare (http://www.metware.cn/) based on the AB Sciex Q-Trap 6500 LC-MS/MS platform. The relative quantitative analysis was performed using a multiple reaction monitoring (MRM) model of triple four-stage mass spectrometry. The peak area of each chromatographic peak represented the relative content of the corresponding substance, which was substituted into the linear equation and calculation formula. Using MultiQuant 3.0.3 software, the qualitative and quantitative analysis results of all of the samples to be measured were finally obtained. R statistical software was used for the differentially accumulated anthocyanins (DAAs), identified and significantly regulated metabolites between groups were determined according to a VIP ≥ 0 and an absolute Log2FC (fold change) ≥ 1.0. Using the KEGG compound database, the annotated metabolites were then mapped to the KEGG pathway database.

### 4.5. RNA Extraction, Quantification, and Sequencing

A total amount of 1 µg of RNA per sample was used as the input material for the RNA sample preparations. The RNA purity was checked using a NanoPhotometer spectrophotometer (IMPLEN, Munich, Germany). The RNA concentration and integrity were determined using the Qubit^®^ RNA Assay Kit in the Qubit^®^2.0 Fluorometer (Life Technologies, Carlsbad, CA, USA) and the RNA Nano 6000 Assay Kit (Agilent Technologies, Santa Clara, CA, USA). A total of 27 libraries were sequenced on the Illumina HiSeq 4000 platform (three biological replicates, respectively), and 125 bp/150 bp paired-end reads were generated [54].

### 4.6. Transcriptome Data Analysis

Fastp v 0.19.3 was used to filter the original data, and all subsequent analyses were based on clean reads. The clean reads were mapped to the *P. serrulate* reference genome using HISAT V2.1.0 (accession number PRJNA1180942). Gene alignment was carried out using featureCounts v1.6.2, and the FPKM of each gene was calculated based on the gene length. We used StringTie v1.3.4d for new gene predictions (Supplementary Materials). DESeq2 v1.22.1 was used to analyze the differential expression genes (DEGs), and genes with a |log2foldchange| ≥ 1 and an FDR < 0.05 were described as DEGs [55]. The GO and KEEG enrichment analyses were performed based on the R 4.3.3 software.

### 4.7. qRT-PCR Validation

A total of nine structure genes and four transcription factors genes related to anthocyanin were selected for validation using quantitative real-time PCR (qRT-PCR). Total RNA was extracted using a UPure Plant RNA Plus Kit (TIANGEN, Beijing, China). The RNA was reverse-transcribed using the Evo M-MLV Mix kit (AIKERUI, Changsha, China). The primers were designed using Primer 3 (https://primer3.ut.ee/) (Table S12) and synthesized by Bioengineering (Shanghai) Co., Ltd., Shanghai, China. The RT-qPCR reactions were performed according to the instructions in the SYBR Green Premix Pro Taq HS qPCR kit (AIKERUI, Changsha, China). PCR amplification was carried out at 95 °C for 30 s, followed by 40 cycles of 95 °C 5 s and 60 °C for 30 s. The relative gene expression was calculated using the 2−ΔΔCt method [56].

## 5. Conclusions

Through qualitative and quantitative analyses of anthocyanins and transcriptomic data, the molecular mechanism of flower color variation was studied. The results showed that the formation of pink and green petals was related to the biosynthesis of anthocyanins and chlorophyll. The content of cyanidin-3-O-glucoside and cyanidin-3-O-(6-O-malonyl-β-D-glucoside) in AR was the highest. A total of 11 differential metabolites were identified to be up-regulated in AR, which were mainly regulated by two structural genes, *DFR* and *UFGT*. By studying the MYB transcription factors, it was found that three transcription factors, *PsMYB77*, *PsMYB1,7* and *PsMYB105*, were grouped together with genes known to positively regulate anthocyanin, which may be involved in the positive regulation of anthocyanin biosynthesis together with the *DFR* and *UFGT* structural genes. These research results are conducive to elucidating the regulatory mechanism of anthocyanin synthesis and the molecular mechanism of the formation of different flower colors and provide a certain scientific basis for the molecular research of cherry flower colors.

## Data Availability

Transcriptome data has been uploaded to the NCBI database: PRJNA1180942. The GenBank accession numbers are, respectively, PsMYB77 (PQ483595); PsMYB17 (PQ483596); PsMYB105 (PQ483597); PsMYB72 (PQ483598); PsMYB86 (PQ483599); and PsMYB45 (PQ483600). The remaining experimental data are provided in Supplementary Materials.

## Supplementary Materials

The following supporting information can be downloaded at https://www.mdpi.com/article/10.3390/plants14010114/s1. Figure S1: Principal component analysis; Figure S2: Differentially expressed gene volcano map of *P. serrulata*; Table S1: Anthocyanin species and content in petals of different varieties of *P. serrulata*; Table S2: Venn diagram of differential metabolites and bar chart of differential metabolites; Table S3: Contents of different metabolites in the anthocyanin biosynthesis pathway of *P. serrulata*; Table S4: Statistics on sequencing data quality from *P*. *serrulata* samples; Table S5: Comparison efficiency statistics for *P. serrulata*; Table S6: All DEG information; Table S7: Different genes between cultivars and differential genes at different developmental stages; Table S8: Differential gene K-means clustering and transcription factor statistics; Tables S9 and S10: Chlorophyll and anthocyanin metabolism heat maps of related genes; Table S11: Phylogenetic tree and differential expression heat maps of related genes and their sequence files; Table S12: Expression primers for anthocyanin-synthesis-related candidate genes; Txt: Phylogenetic tree for all Myb proteins.
