# Supplementary figures and images for: Integrated Metabolomics and Transcriptome Analysis of Anthocyanin Biosynthetic Pathway in Prunus serrulata
Source: Plants (Basel). 2025 Jan 3;14(1):114. doi: 10.3390/plants14010114 (PMC11723355; doi:10.3390/plants14010114)

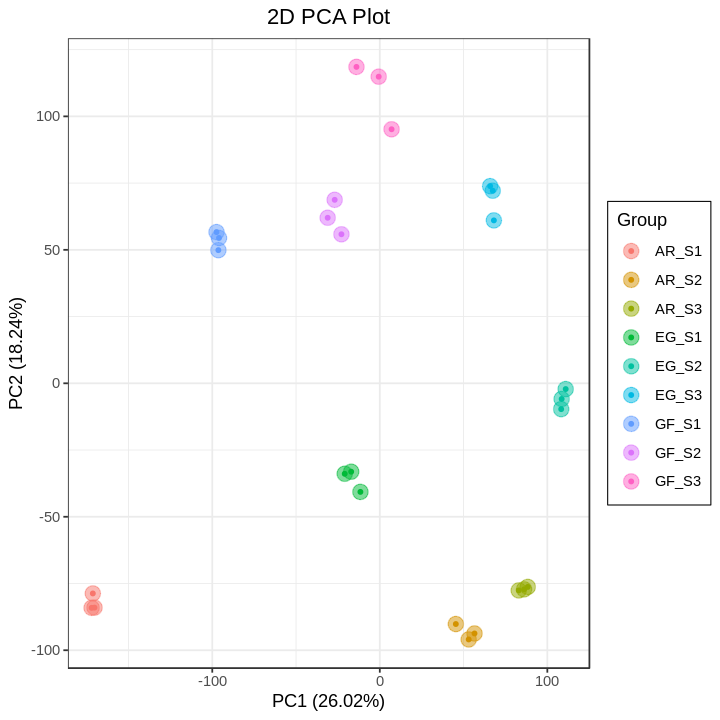

Supplement: Supplementary file 1 [file plants-14-00114-s001.zip › FigureS1-Principal component analysis.png]

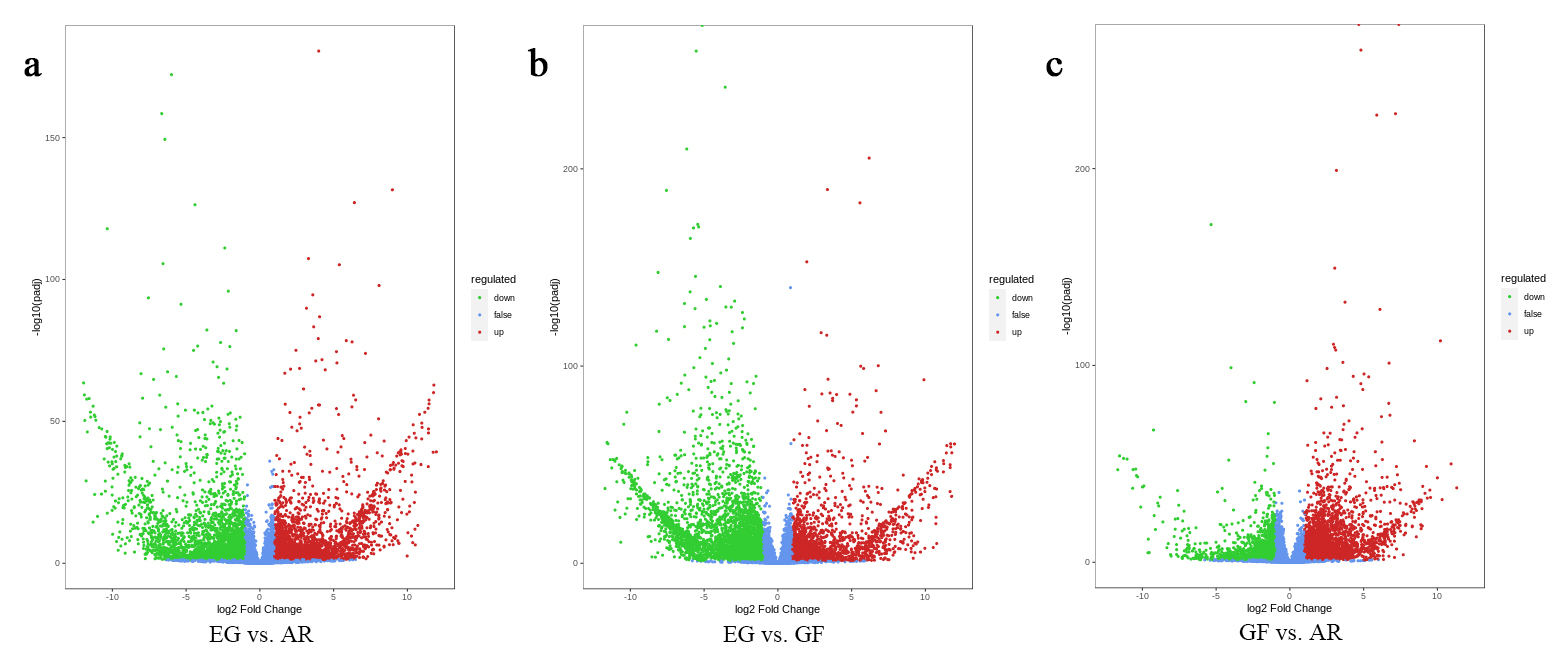

Supplement: Supplementary file 1 [file plants-14-00114-s001.zip › FigureS2-Differential expressed gene volcano map of P. serrulata.jpg]
